# Effects of Ethanol Extract from Senna Leaf (EESL) on Inflammation and Oxidative Stress in Mice: A Non-targeted Metabolomic Study

**DOI:** 10.2174/0118715303325372241014152811

**Published:** 2024-10-24

**Authors:** Xiaoli Huang, Wen Sun, Chang Sun, Jiajun Tan, Liang Wu, Fumeng Yang

**Affiliations:** 1 Medical Laboratory Department, Sihong Hospital, Sihong, China;; 2 Department of Critical Care Medicine, Jurong Hospital, Affiliated to Jiangsu University, Zhenjiang, China;; 3 Department of Laboratory Medicine, School of Medicine, Jiangsu University, Zhenjiang, China;; 4 Department of Laboratory Medicine, Lianyungang Second People's Hospital Affiliated to Jiangsu University, Lianyungang, China

**Keywords:** Ethanol extract of *Senna* leaf (EESL), oxidative stress, inflammation, intestinal mucosa, tight junction, drug dependence

## Abstract

**Background:**

Senna leaf is a commonly used medication for treating constipation, and long-term use can cause damage to the intestinal mucosa and lead to drug dependence. But the exact mechanism remains unclear.

**Objective:**

Using non-targeted metabolomics technology to study the mechanism of senna leaf ethanol extract (EESL) inducing inflammation and oxidative stress in mice and causing side effects.

**Methods:**

EESL was administered to mice by gavage to detect inflammation and oxidative stress-related factors in mice, and the EESL components and differential metabolites in mouse plasma were analyzed using non-targeted metabolome techniques.

**Results:**

23 anthraquinone compounds were identified in the EESL, including sennoside and their derivatives. Administration of EESL to mice resulted in a significant increase in pro-inflammatory factors, IL-1β, and IL-6 in the plasma, while the levels of IgA significantly decreased. The levels of oxidative stress significantly increased, and the intestinal mucosal integrity was impaired. 21 endogenous in plasma metabolites were identified as differential metabolites related with taurine and taurine metabolism, glycerophospholipid metabolism, arachidonic acid metabolism, tryptophan metabolism, and sphingolipid metabolism. These metabolic pathways are related to oxidative stress and inflammation.

**Conclusion:**

Senna leaf can inhibit the expression of tight junction proteins in the intestinal mucosa and disrupt intestinal mucosal barrier integrity, exacerbating oxidative stress and inflammation induced by bacterial LPS entering the bloodstream. In addition, the impact of Senna leaf on tryptophan metabolism may be linked to the occurrence of drug dependence.

## 1 INTRODUCTION

*Cassia angustifolia* Vahl is a leguminous plant called Senna, and Senna leaf is a commonly used cathartic drug. It can directly act on intestine, enhance the peristalsis of the large intestine, and make constipation patients defecate in a short time [1]. Senna leaf is an effective remedy for intractable constipation [2]. However, clinical studies have also found that long-term use of Senna leaf in the treatment of constipation can easily lead to drug dependence, digestivetract bleeding, and even intestinal mucosa damage [3-5]. The dependence symptoms of Senna leaf are observed in habitual constipation patients who use Senna leaf for treatment [6]. The withdrawal symptoms are similar to the early symptoms of opioid drug dependence, mainly manifesting as anxiety, restlessness, body aches, insomnia, loss of appetite, increased blood pressure, and weight loss [7, 8]. Other studies have shown that when the amount of senna leaf brewed with boiling water is greater than 10 g-30 g, symptoms of upper gastrointestinal bleeding may occur [9, 10].

The main components of Senna leaf are anthraquinones and their derivatives [11]. It is believed that they can improve the structure of intestinal flora and the formation of metabolites, and can cause damage to the intestinal mucosa [12, 13]. Due to the destruction of the intestinal mucosal barrier, a large amount of lipopolysaccharides from intestinal bacteria are released into the blood, triggering systemic chronic inflammation [14]. It is well known that chronic inflammation is a major cause of various chronic diseases, and long-term low-grade chronic inflammation is extremely harmful to health [15]. Therefore, studying the mechanism of how Senna leaf destroys the intestinal mucosal barrier and induces systemic chronic inflammation is of great significance for the use of Senna leaf.

In this study, we investigated the effects of oral EESL on mouse intestinal mucosal injury, *in vivo* inflammatory and oxidative stress response levels, as well as the impact on plasma metabolic profiling, using a mouse model. We aimed to explore the mechanism underlying the side effects of Senna leaf.

## 2 MATERIALS AND METHODS

### 2.1 Chemicals and Reagents

Senna leaf in this study was purchased from Bozhou Mingjie Biotechnology Co., LTD (Bozhou, Anhui, China). To prepare the ethanol extract from the Senna leaf, the method reported by Ontong *et al.* was followed [8]. The operation steps were as follows: 2 g coarse Senna leaf powder was precisely weighed and placed in a round-bottomed flask, and 20 mL 50% ethanol was added and heated for reflux for 15 min. The above operation was repeated three times, and the obtained liquid was filtered by 0.45 μm filter membrane, and then the ethanol extract of senna leaf (EESL) was obtained by vacuum rotary evaporation. Each ml of the above ethanol extract contains 0.2 g senna components. The EESL components were analyzed by UPLC-MS technique. The analysis of UPLC-MS was performed by Wekemo Tech Group Co., Ltd. (Shenzhen, China).

### 2.2 Animal Treatment and Sample Collection

The male ICR mice (weighing 18-22 g) were provided by Wukong Biotechnology Co., Ltd. (Nanjing, China). The study was conducted in accordance with the National Institutes of Health guide for the care and use of Laboratory animals (NIH Publications No. 8023, revised 1978), and approved by the Ethics Committee of Jiangsu University (protocol code UJS-IACUC-AP-2021022804 and date of approval: January 2021).

In this study, the mice were divided into three groups including the normal control group (NC group), the low concentration of EESL treated group (FL group) and the high concentration of EESL group (FH group) with 10 mice in each group. All mice were held under standard laboratory conditions, fed with Lab Rat and Mouse Diet from Xietong Biotechnology Co., Ltd. (Nanjing, China), the temperature was (25 ± 1)°C, the humidity was (50 ± 10)%, and dark/light 12 h/12 h cycle.

The mice in the NC group were given 200 μL sterilized distilled water by gavage daily, and those in the FL and FH groups were given 20 μL and 200 μL EESL by gavage daily for 15 days. Mice were sacrificed on the 16th day, and plasma was collected for subsequent study.

### 2.3 Lipolyaccharide (LPS) Level Detection

The LPS detection in mouse plasma was determined using the BET-24A bacterial endotoxin analyzer employing the dynamic turbidimetric limulus reagent method. The kit utilized for this analysis was provided by Xiamen Limulus Reagent Biotechnology, based in Xiamen, China. The instructions provided with the kit were meticulously followed.

### 2.4 Oxidative Stress and Inflammatory Factor Detection

The levels of plasma pro-inflammatory cytokines including IL-1β and IL-6, as well as the indicators of mucosal defense of IgA were detected by ELISA assay. The ELISA kits were purchased from Jiangsu Meimiang Industry (Yanchen, China). After the reaction, the absorbance was measured at 450 nm, and the concentration of each cytokine was calculated according to the standard curve. The levels of relevant indicators in mouse plasma were detected using the malondialdehyde (MDA) reagent kit and superoxide dismutase (SOD) reagent kit from Nanjing Jiancheng Bioengineering Institute (Nanjing, China). The mouse reactive oxygen species cluster (ROS) ELISA kit from Jining Shiye Bioengineering Institute (Shanghai, China). The detection procedures were strictly conducted according to the instructions of the respective reagent kits.

### 2.5 Non-targeted Metabolomics Analysis

The mouse plasma of the NC and FH groups stored at -80°C was thawed at 4°C for non-targeted metabolomics analysis. Non-targeted metabolomics detection was performed by Wekemo Tech Group Co., Ltd. (Shenzhen, China). The data were analyzed on the free online platform of Wekemo Bioincloud (https://www.bioincloud.tech/) including the unsupervised principal component analysis (PCA) and orthogonal least squares discrimination analysis (OPLS-DA). Variable importance in the projection (VIP) reflects the contribution of the analyzed variables to the OPLS-DA model. The differential metabolites were screened according to the criteria of VIP > 1 and *P* < 0.05. These differential metabolites were identified using mass spectroscopic data, which were retrieved and confirmed in the Human Metabolome Database (HMDB, http://www.hmdb.ca/). Kyoto Encyclopedia of Genes and Genomes Database (KEGG, http://kegg.jp/kegg/kegg1.html) was used for the detection of metabolic pathways associated with potential differential metabolites.

### 2.6 HE Staining and Immunohistochemistry of Mouse Jejunum Tissue

Intestine tissue was obtained 10 cm from the blind part of the mouse, fixed with 4% paraformaldehyde, dehydrated, embedded in paraffin, and stained with hematoxylin and eosin after sectioning. The morphological changes of the intestinal mucosa were observed under a microscope. In the immunohistochemical staining of tight junction proteins ZO-1 and Occludin, the paraffin sections of the intestinal tissue were dewaxed and washed three times with pH 7.4 PBS buffer. A citric acid antigen retrieval solution was used to block endogenous peroxidase activity in the tissue samples. The primary antibody was added and incubated at room temperature for 2 hours, followed by DAB staining and counterstaining with hematoxylin. The slides were then sealed with neutral gum and observed under a microscope.

### 2.7 Statistical Analysis

Results were expressed as mean ± standard deviation. SPSS 22.0 statistical software was used for statistical analysis; the one-way ANOVA test was used to test the overall mean; the LSD-t method was used to test the multiple comparisons of sample means between groups. *P* <0.05 was considered statistically significant and Graphpad Prism 9.2 was used for mapping.

## 3 RESULTS

### 3.1 Analysis of EESL Components by UPLC-MS

**Fig. (1):**
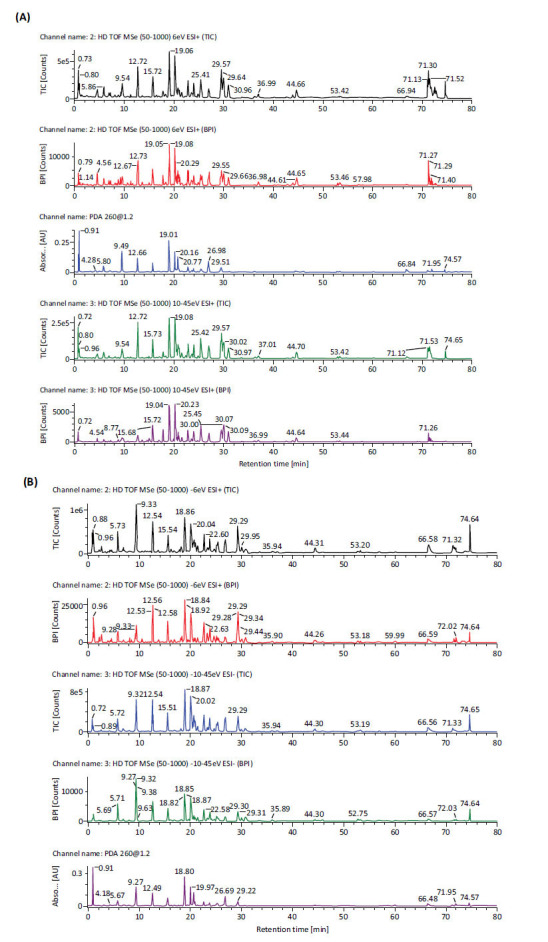
Analysis of ethanol extract components of senna leaf (EESL) based on UPLC-MS technique. **A**: Total ion chromatography (TIC) in positive ion mode (ESI^+^); **B**: Total ion chromatography (TIC) in negative ion mode (ESI^-^).

The EESL component analysis was performed by Wekemo Tech Group Co., Ltd. (Shenzhen, China). For a more comprehensive analysis of the compounds in EESL, total ion chromatograms (TICs) obtained in both positive and negative modes were compared in this study. Twenty-three components were identified in EESL by UPLC-Q-TOF-MS, including sennoside A, sennoside B, sennoside C and sennoside D (Fig. **1** and Table **1**).

### 3.2 EESL Increased Plasma Inflammatory Factors and Disruption of Mucosal Barrier in Mice

**Fig. (2):**
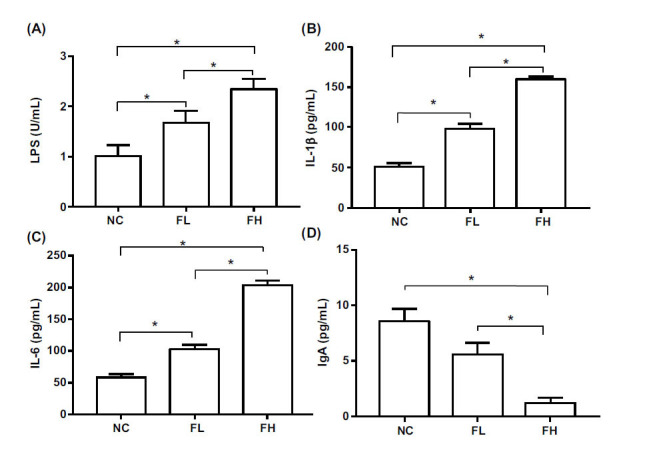
Expressions of pro-inflammatory cytokines and IgA by ELISA assay. The ICR mice were pretreated with 20 μL and 200 μL EESL. **A**: LPS level; **B**: IL-1β level; **C**: IL-6 level; **D**: IgA level. After treated with the high concentration and low concentration of EESL, the concentrations of inflammatory factors LPS, IL-1β and IL-6 were decreased, and mucosal immunity index IgA was decreased. It was concentration-dependent (*: *P* < 0.05, n=3).

The concentration of LPS in the FL and FH groups was significantly increased after oral administration of EESL compared with the NC group (*P* <0.05); there was a positive correlation between plasma LPS level and the dosage of EESL (Fig. **2A**).

Compared to the NC group, the expressions of IL-1β and IL-6 were significantly increased (*P* <0.05); compared to the FL group, the expressions of IL-1β and IL-6 were significantly increased (*P* <0.05) (Fig. **2B** and **2C**). The level of plasma IgA in the FL and FH groups significantly decreased (*P* < 0.05) compared with the NC group; moreover, IgA level in the FL group was significantly higher than in the FH group (*P* > 0.05) (Fig. **2D**).

### 3.3 EESL Enhance Oxidative Stress Response in Mice

**Fig. (3):**
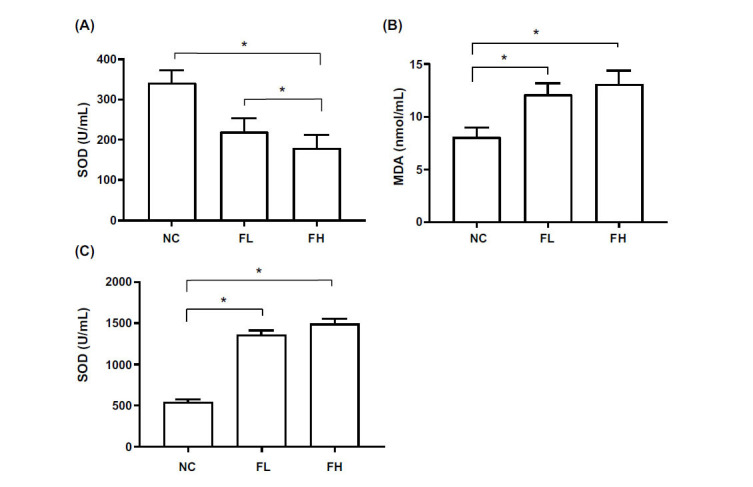
SOD and MDA levels in plasma of mice. The ICR mice were pretreated with 20 μL and 200 μL EESL. **A**: SOD level; **B**: MDA level; **C**: ROS level. After treated with the high dose and low dose of EESL, the level of oxidative stress in mice was significantly increased. The SOD levels decreased significantly, MDA and ROS levels increased significantly (*: *P* < 0.05, n=3).

Compared with the NC group, the levels of SOD in the plasma of the FL and FH groups of mice were significantly decreased (*P* < 0.05), but there was no significant difference between the FL and FH groups (*P* > 0.05). The levels of MDA and ROS in the plasma of FL and FH groups of mice were significantly increased (*P* < 0.05), but there was no significant difference between the FL and FH groups (*P* > 0.05) (Fig. **3**).

### 3.4 EESL Damaged the Tight Junction Structure of Intestinal Mucosa in Mice

**Fig. (4):**
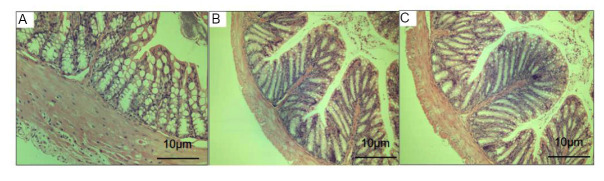
HE staining of mouse intestinal mucosa. **A**: NC group; **B**: FL group; **C**: FH group. The intestinal mucosal structure of mice in NC group was normal. After EESL treatment, obvious necrosis of intestinal mucosal cells and loss of mucosa can be seen, and there was significant dilation and congestion of the submucosal capillaries, with a small amount of neutrophil infiltration in the stroma. There was no significant difference between the FL and FH groups.

After HE staining of the small intestine of experimental mice, it can be observed that the intestinal mucosa of the NC group mice were intact and the cells were arranged neatly, with only a small amount of cell necrosis and shedding. In the FL and FH groups, obvious necrosis of intestinal mucosal cells and loss of mucosa can be seen, and there was significant dilation and congestion of the submucosal capillaries, with a small amount of neutrophil infiltration in the stroma. There was no significant difference between the FL and FH groups (Fig. **4**).

**Fig. (5):**
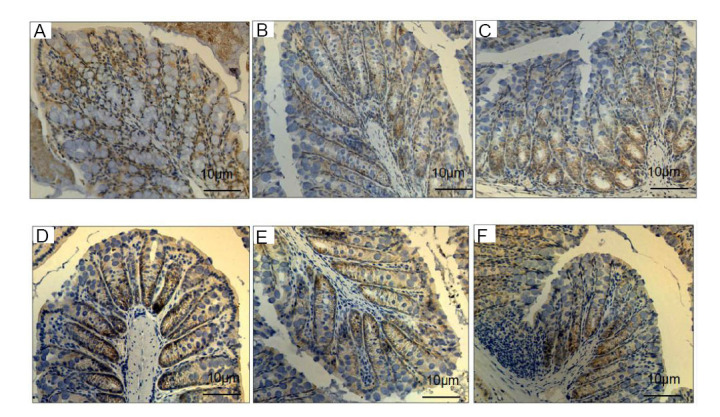
Immunohistochemical staining of ZO-1 and Occludin in mouse intestinal mucosa. **A**: ZO-1 expression in the NC group; **B**: ZO-1 expression in the FL group; **C**: ZO-1 expression in the FH group; **D**: Occludin expression in the NC group; **E**: Occludin expression in the FL group; **F**: Occludin expression in the FH group. After immunohistochemical staining, the tight junction proteins ZO-1 and Occludin expression site showed brownish-yellow. ZO-1 and Occludin were distributed along the surface of the intestinal epithelial cells, with no expression in the cell nuclei and nuclear membranes. After EELS treatment, the expressions of ZO-1 and Occludin on the surface of the intestinal epithelial cells were significantly reduced, and some of the surface expression was completely absent. The expressions of ZO-1 and Occludin were concentration-dependent.

Immunohistochemical staining showed that in the NC group, the tight junction proteins ZO-1 and Occludin were distributed along the surface of the intestinal epithelial cells, appearing brownish-yellow, with no expression in the cell nuclei and nuclear membranes. In the FL and FH groups, the ZO-1 and Occludin proteins on the surface of the intestinal epithelial cells were significantly reduced, and some of the surface expression was completely absent; the ZO-1 and Occludin proteins on the surface of the intestinal epithelial cells were further reduced compared to the FL group (Fig. **5**).

### 3.5 EESL Regulates Plasma Metabolites in Mice

**Fig. (6):**
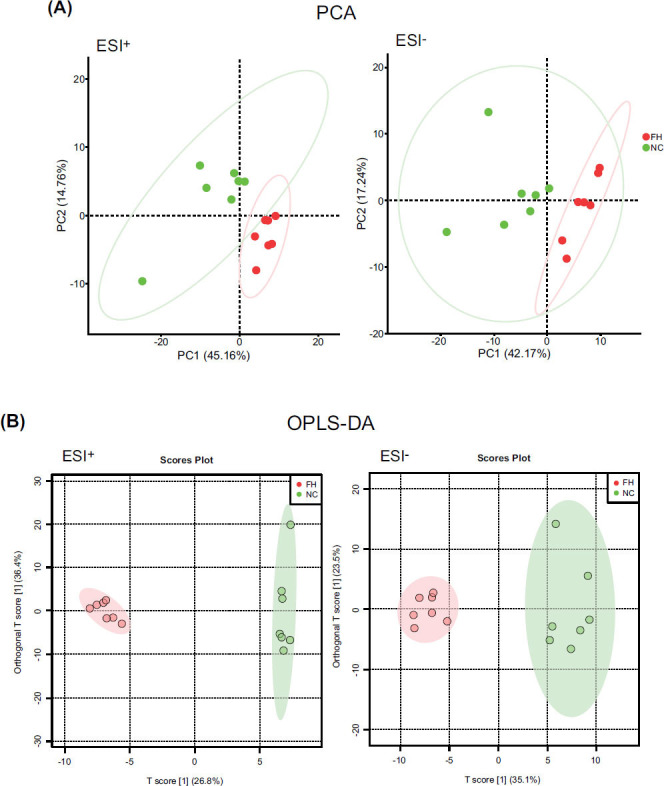
PCA score plot and OPLS-DA score plot in the ESI^+^ and ESI^-^ modes analysis (n = 7). **A**: PCA score plot analysis in the ESI^+^ and ESI^-^ modes in NC and FH groups; **B**: OPLS-DA score plot analysis in the ESI^+^ and ESI^-^ modes in the NC and FH groups. The samples of the FH and NC groups could be separated and clustered respectively under the ESI^+^ and ESI^-^ modes, indicating that the metabolomics of mice treated with EESL had significant changes.

**Fig. (7):**
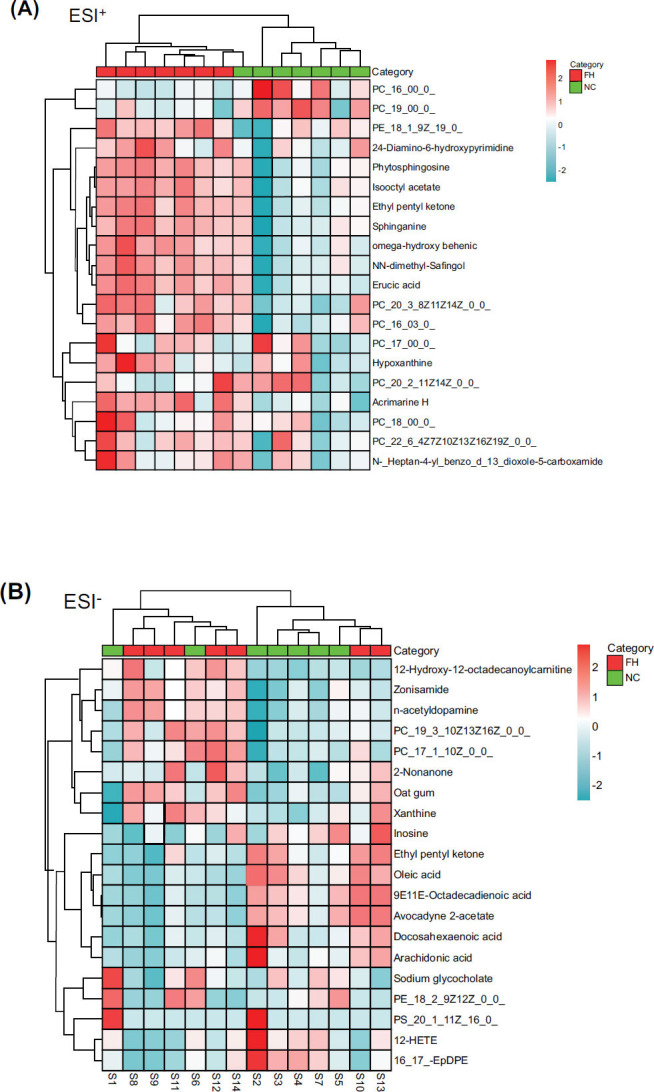
The heat maps of differential metabolites analysis. Rows: metabolites, columns: samples. On the top is the cluster of samples, and on the left is the cluster of metabolites. Red means the metabolites were expressed at a higher level, and blue means the metabolites were expressed at a lower level. **A**: the ESI^+^ mode; **B**: the ESI^-^ mode. Heat map results showed that there were significant changes in plasma concentrations of several metabolites in the FH and NC groups.

From the PCA scatter plot and OPLS-DA scatter plot, it can be seen that the samples of the NC and FH groups were completely separated in the ESI^+^ and ESI^-^ modes (Fig. **6**). This suggests that EESL can significantly affect plasma metabolomics in mice. It can be seen from the clustering heat map that metabolites in plasma samples of the NC and FH groups differ markedly (Fig. **7**).

In the ESI^+^ mode, the plasma levels of hydroxybutyric acid, acetic acid, *L*-acetyl-carnitine, 7α-hydroxy-3-oxo-4-cholestenoate, (*E*)-2-butenal, creatine, indoles, betaine and niacinamide in the FH group increased if compared with the NC group. However, phosphatidyl choline reduced flavin mononucleotide, ceramide, phosphatidyl ethanolamine, tetrahydrodeoxycorticosterone, 17α,21-dihydroxypregnenol- one, 4-oxy-retinol, retinoid, enolone, 8,11,14-eicosatrienoic acid and *m*-coumaric acid decreased in the FH group (Table **2**).

In the ESI- mode, compared with the NC group, phosphatidyl ethanolamine, cysteine sulfonic acid, apigenin, gamma-*L*-glutamyl taurine, 15(*S*)-hydroxyeicosanotrienoic acid, monohydroxy-bile acid, *L*-leucine, 3-(3-hydroxyphenyl) propionic acid, phosphatidylcholine, taurine, vitamin E, norepinephrine, indolealdehyde, hypoxanthine 2,3-diaminopropionic acid, xanthine, and deoxycorticosterone increased in the plasma of mice in FH group, but arachidonic acid, 5'-pyridoxine phosphate, *n*-oleylethanolamine, tryptophan, epinephrine, xanthine nucleoside, dihydroisoflavone, dihydrocortisol, enkephalin, 12-hydroxy-eicosanotetraenoic acid, and ceramide decreased (Table **2**).

**Fig. (8):**
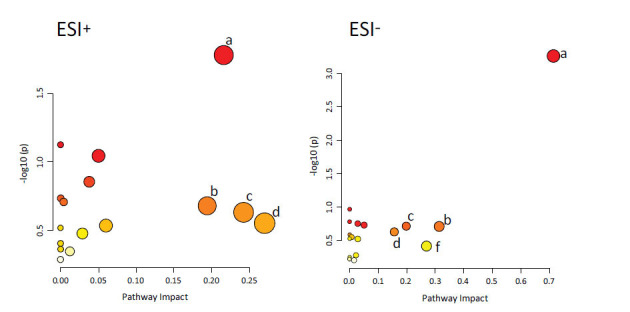
EESL regulates metabolic pathways in mice. a: Glycerophospholipid metabolism; b: Arachidonic acid metabolism; c: Glycerophospholipid metabolism; d: Tryptophan metabolism; f: Sphingolipid metabolism. The regulation of EESL on mouse plasma metabolism involves several pathways, including glycerophospholipid metabolism, arachidonic acid metabolism, glycerophospholipid metabolism, tryptophan metabolism and sphingolipid metabolism.

The potential differential metabolites were imported into the KEGG database for enrichment analysis. The results showed that EESL could affect multiple metabolic pathways in mice, including taurine and taurine metabolism, glycerol phospholipid metabolism, arachidonic acid metabolism, tryptophan metabolism, sphingolipid metabolism (Fig. **8**).

## 4 DISCUSSION

Senna Leaf is a commonly used herbal remedy for constipation [9, 16]. Many users believe that Chinese herbs are safe, but this belief is not incorrect. Our study found that oral EESL increased the concentration of bacterial exotoxins in the blood of mice. In this study, we first found that EESL can increase the concentration of Lipopolysaccharides (LPS) in mouse plasma. LPS are primarily derived from gram-negative bacteria in the host gut and have strong pro-inflammatory ability [14, 17]. We speculated that EESL can damage the integrity of the mucosal barrier, and intestinal bacterial LPS can enter the bloodstream through the intestinal mucosa to further induce an inflammatory response. Our immunohistochemical results also confirmed that EESL could inhibit the expression of intestinal mucosal barrier proteins ZO-1 and Occludin in mice. Excessive use of Senna leaves can cause disruption of the intestinal flora, it may also increase the number of gram-negative bacteria, that is, increase the production of LPS [18, 19]. We speculate that oral administration of EESL may disrupt the normal structure of the intestinal flora and increase the number of harmful gram-negative bacteria in the intestines, thereby increasing the concentration of the main inflammatory factor LPS in gram-negative bacteria, causing damage to the intestinal mucosa and further triggering systemic inflammation.

In this study, the plasma levels of proinflammatory cytokines such as IL-1β in EESL treated mice were significantly increased. IL-1β and IL-6 are important pro-inflammatory cytokines, which can induce inflammatory tissue damage [20]. We believe that the increased levels of pro-inflammatory cytokines IL-1β and IL-6 in mouse plasma may be the result of LPS-induced inflammation. The high levels of pro-inflammatory cytokines in the plasma not only reflect immune damage to the mouse intestinal mucosa, but also indicate a systemic high-level inflammatory response [21]. Prolonged systemic inflammation can trigger various chronic diseases, such as aging, non-alcoholic fatty liver disease, and chronic obstructive pulmonary disease [22, 23]. Therefore, it is necessary for patients who use senna leaf for a long time to pay attention to the levels of pro-inflammatory cytokines in their own plasma. IgA plays an important role in host mucosal immunity [24, 25]. The observed effects may be related to the damage of the mucosal barrier, and the change that helps the proinflammatory substances of gut bacteria to enter the blood circulation and induce an inflammatory response [26].

ROS, SOD and MDA are important factors in the oxidative stress response, and the essence of the oxidative stress response is the imbalance between oxidation and antioxidant systems in cells [27, 28]. In this study, the plasma oxidative stress level of EESL treated mice was significantly increased. When the organism or cells are stimulated, a large amount of oxygen free radicals are produced, leading to tissue damage [29]. MDA is the end product of lipid peroxidation of polyunsaturated fatty acids and is involved in lipid oxidation [30]. It is one of the most commonly used biomarkers for evaluating oxidative stress response [31]. SOD is one of the main members of the antioxidant enzyme system in the organism and is a cytoplasmic low-molecular-weight metal protein that can reduce reactive oxygen species to reduce the degree of cell damage [32, 33]. The increase in LPS levels in the blood after EESL administration in mice was the main cause of enhanced oxidative stress response in the mice. Oxidative stress and inflammation were closely related and mutually promote each other, leading to intestinal mucosal damage in mice [34].

The results of mice plasma metabolomics showed that the plasma metabolism of arachidonic acid, tryptophan and glycerophospholipid significantly changed. Arachidonic acid is one of the most widely distributed and biologically active omega-6 polyunsaturated fatty acids [35]. Arachidonic acid binds mainly to phosphatidyl choline or phosphatidyl ethanolamine glycerol and is present in cell membranes [36]. *In vivo* phospholipase A2 (PLA2) is activated when an inflammatory substance (*e.g.,* LPS) intrudes into an organism, catalyzing the hydrolysis of phospholipid diacylates, resulting in the decomposition of arachidonic acid into free forms for release into cytoplasm [37, 38]. Free arachidonic acid, when combined with free 5-lipoxyase activator protein, can be catalyzed by 5-lipoxyase into intermediate eicosanotetraenoic acid 5-hydroxyperoxide, which is then oxidized to leukotriene A4 and 5-hydroxyeicosanotetraenoic acid [39, 40]. Leukotriene is a highly potent chemokine of inflammatory cells, which can also induce inflammatory response [41].

We also found that the content of hydroxybutyric acid, acetic acid, 7α-hydroxy-3-oxo-4-cholestenoate, indoles, betaine and niacinamide increased in mice plasma, suggesting that this was a stress response to inflammation *in vivo*. Hydroxybutyric acid and acetic acid belong to short-chain fatty acids, which are anti-inflammatory substances obtained from intestinal flora fermentation of indigestible dietary fiber, and have a wide range of anti-inflammatory functions [42-44]. Betaine is an amino acid derivative that is not only ingested by humans in food, but also synthesized in liver and kidneys as endogenous betaine [45]. An important function of betaine is to suppress the expression of inflammatory factors, scavenge free radicals, and act as an antioxidant, which can effectively inhibit the inflammatory response in live organisms [46, 47]. Indole is the metabolite of tryptophan in human gut [48]. Some cells in the gut can metabolize tryptophan into indole derivatives, which are ligands of aryl hydrocarbon receptor (AHR) [49, 50]. AHR can affect cellular signaling by interacting with various regulatory signaling proteins, including PAS heterodimer chaperones ARNT (aromatics receptor nuclear transporters), immune-like proteins such as heat shock protein-90, AIP, p23, protein kinases, and phosphokinases (*e.g.,* tyrosine kinases, CK2, PKC, *etc.*) [51, 52]. AHR also interacts with signaling pathway mediated by ESR (estrogen receptor) and other hormone receptors, such as hypoxia, NF-κB, and Rb proteins [53].

Tryptophan and its metabolites are currently a hot topic of research in the prevention and alleviation of drug dependence and addiction [54, 55]. Patients who use Senna leaves for long-term treatment of constipation may also experience drug dependence, but the exact mechanism is not clear [56]. In this study, we noticed a significant decrease in tryptophan levels in the plasma of mice after oral administration of Senna leaves. Tryptophan is an essential amino acid that cannot be synthesized by the human body and must be obtained from food [57]. It plays a crucial role in various metabolic processes in the human body, including promoting muscle development and enzyme function, as well as regulating the production and physiological effects of various neurotransmitters in the body [58, 59]. Tryptophan is a precursor to metabolites in the serum, including serotonin, melatonin, and kynurenine [60]. Most of the daily intake of tryptophan is oxidized through the kynurenine pathway, while the rest is degraded through the serotonin pathway [61-63]. Tryptophan is first converted into 5-hydroxytryptophan, which is then metabolized into serotonin by different enzymes, and further metabolized into melatonin [64, 65]. The kynurenine pathway involves the generation of kynurenic acid (KA) and the formation of 3-hydroxyanthranilic acid, and is involved in the further synthesis of quinolinic acid (QA) [66]. It is currently believed that both QA and KA have significant effects on neurons in the central nervous system, with QA being a potential neurotoxin and KA being a neuroprotective agent [67, 68]. 3-hydroxykynurenine is a third metabolite of kynurenine, which may generate free radicals and exacerbate neuronal damage [69, 70]. We speculate that the regulation of tryptophan metabolism by Senna leaves may be a potential cause of addiction, and whether supplementation with tryptophan can prevent and treat Senna leaf dependence is worthy of further in-depth study in the next stage.

## CONCLUSION

In summary, we believe that Ethanol Extract of Senna Leaf (EESL) can disrupt intestinal mucosal integrity, leading to the entry of bacterial lipopolysaccharides (LPS) into the bloodstream and triggering systemic inflammatory and oxidative stress reactions. We believe that patients who use Senna leaf for a long period of time should regularly test indicators such as LPS and SOD in their blood to avoid chronic inflammatory reactions caused by LPS from the intestines entering the bloodstream. The reason for patients developing drug dependence on Senna Leaf may be related to its regulation of plasma tryptophan metabolism. Supplementing exogenous tryptophan may be an effective method for preventing drug dependence, but further research is still needed.

## Figures and Tables

**Table 1:** Components identified in ethanol extract from senna leaf (EESL).

Peak No.	*t*_R_ (min)^a^	Molecular formula	Observed (*m*/*z*)	MS^2^ ions (*m*/*z*)	Identification
1	0.88	C_19_H_16_O_4_	307.09775 (M-H)	175.04898, 157.03708	Didemethoxycurcumin
2	0.96	C_20_H_18_O_5_	337.09073 (M-H)	307.09775, 175.04898, 157.03708	Demethoxycurcumin
3	1.61	C_15_H_20_O_10_	383.09862 (M+Na)	245.06558, 199.06010	Glucosyringic acid
4	5.73	C_25_H_22_O_9_	465.12413 (M-H)	285.05292, 241.06130, 224.05689	Kaempferol derivative
5	6.83	C_29_H_38_O_16_	641.16159 (M-H)	441.15922, 353.10197, 299.03212, 255.04088	Hydroxy-rhein derivative
6	9.13	C_23_H_21_O_10_	457.15489 (M-H)	387.18168, 185.02920, 163.11865, 119.05476	Anthraquinones derivative
7	15.54	C_27_H_30_O_17_	627.16700 (M+H)	465.11231, 303.05564, 229.05411, 153.02038	Quercetin-3-*O*-gentiobioside
8	18.87	C_27_H_30_O_16_	609.17277 (M-H)	285.05292, 255.04088, 229.06047	Rutin
9	20.04	C_28_H_32_O_17_	639.18480 (M-H)	315.06356, 300.03967, 285.05292, 271.03654, 255.04088, 243.04048	Isorhamnetin-3-*O*-gentiobioside
10	20.44	C_42_H_40_O_19_	847.24651 (M-H)	685.18401, 641.19370, 545.10708, 431.11530, 386.11676, 224.05689	Sennoside C
11	20.65	C_42_H_38_O_20_	861.22167 (M-H)	699.16529, 655.17221, 610.17178, 559.08668, 537.10288, 431.11530, 386.11676, 269.05651, 224.05689	Sennoside B
12	20.89	C_42_H_40_O_19_	847.24651 (M-H)	685.18401, 641.19049, 545.10708, 479.13189, 431.11530, 386.11676, 341.07979, 269.05651, 224.05689	Sennoside D
13	21.39	C_28_H_34_O_15_	609.17277 (M-H)	479.12654, 317.07126, 270.05650, 153.02038	Neohesperidin
14	22.60	C_27_H_30_O_15_	593.17638 (M-H)	285.05292, 255.04088, 227.04362	Kaempferol-3-*O*-rutinoside
15	23.26	C_21_H_20_O_11_	447.111181 (M-H)	285.05078, 255.04088, 227.04362	Kaempferol-3-*O*-glucoside
16	23.48	C_42_H_40_O_19_	847.24282 (M-H)	685.18401, 386.11676, 224.05689	Sennoside derivative
17	23.74	C_28_H_32_O_16_	623.18905 (M-H)	315.06356, 300.03967, 271.03654	Narcissoside
18	24.58	C_22_H_22_O_12_	477.12399 (M-H)	431.11530, 315.06131, 299.03212, 285.05292, 271.03654, 243.04048	Isorhamnetin-3-*O*-glucoside
19	25.14	C_42_H_40_O_19_	847.24282 (M-H)	685.18401, 386.11676, 224.05689	Sennoside derivative
20	25.36	C_42_H_38_O_20_	861.22167 (M-H)	699.16194, 677.52644, 431.11530, 386.11676, 269.05651, 224.05689	Sennoside derivative
21	26.78	C_42_H_38_O_20_	861.22167 (M-H)	699.15629, 386.11676, 224.05689	Sennoside A
22	29.29	C_20_H_24_O_9_	407.15105 (M-H)	245.09128, 230.06744, 215.07954, 201.06376, 199.04738	Tinnevellin glycoside
23	30.85	C_27_H_30_O_15_	593.17638 (M-H)	269.05651, 225.06422	Emodin-1-*O*-β-gentiobioside

**Table 2:** The differential metabolites between the NC and FH groups.

Metabolite	HMDB ID	KEGG ID	*m*/*z*	*t*_R_ (min)	*vs.* NC group
ESI+					
2-Hydroxybutyric acid	HMDB0000008	C05984	231.08	1.73	↑
PC(16:0/0:0)	HMDB0010382	C04230	518.32	6.27	↓
Acetic acid	HMDB0000042	C00033	98.98	1.09	↑
*L*-Acetylcarnitine	HMDB0000201	C02571	204.12	1.36	↑
Tetrahydrodeoxycorticosterone	HMDB0000879	C13713	373.23	7.52	↓
Methoxamine	HMDB0014861	C07513	229.15	0.97	↑
7-Alpha-hydroxy-3-oxo-4-cholestenoate	HMDB12458	C17337	431.31	6.15	↑
Indole	HMDB0000738	C00463	118.07	3.14	↑
Betaine	HMDB0000043	C00719	118.09	0.86	↑
Niacinamide	HMDB0001406	C00153	123.06	1.37	↑
ESI-					
Arachidonic acid	HMDB0001043	C00219	683.59	23.00	↑
PE(18:2(9Z,12Z)/22:4(7Z,10Z,13Z,16Z))	HMDB0009108	C00350	836.55	6.05	↑
Tryptophanol	HMDB0003447	C00955	206.08	4.41	↓
15(*S*)-Hydroxyeicosatrienoic acid	HMDB0005045	C00861	321.24	7.15	↑
7α-hydroxy-3-oxo-4-cholestenoate	HMDB12458	C17337	429.30	6.15	↑
*L*-Leucine	HMDB0000687	C00123	130.09	1.92	↑
Enkephalin L	HMDB0001045	C16041	554.26	0.01	↓
Hypoxanthine	HMDB0000157	C00262	271.07	3.15	↑
12-HETE	HMDB0006111	C14777	319.23	6.76	↓
Deoxycorticosterone	HMDB0000016	C03205	311.20	7.70	↑

## Data Availability

The data and supportive information is available within the article.

## LIST OF ABBREVIATIONS

AHR: Aryl Hydrocarbon Receptor
ARNT: Aromatics Receptor Nuclear Transporters
EESL: Ethanol Extract of Senna Leaf
KA: Kynurenic Acid
LPS: Lipopolysaccharides
PLA2: Phospholipase A2
QA: Quinolinic Acid

## References

[1] Wu C., Zheng K., Meng T., Wang J. (2020). Effects of endovascular stent-assisted effects of various frequencies of abdominal naprapathy on changes in gastrointestinal mucosal cells in spleen-deficient rabbits.. Med. Sci. Monit..

[2] Rao S.S.C., Brenner D.M. (2021). Efficacy and safety of over-the-counter therapies for chronic constipation: An updated systematic review.. Am. J. Gastroenterol..

[3] Zhu J., Li X., Deng N., Peng X., Tan Z. (2022). Diarrhea with deficiency kidney-yang syndrome caused by adenine combined with *Folium senna* was associated with gut mucosal microbiota.. Front. Microbiol..

[4] Kiliś-Pstrusińska K., Wiela-Hojeńska A. (2021). Nephrotoxicity of herbal products in Europe-A review of an underestimated problem.. Int. J. Mol. Sci..

[5] Coskun Y., Yuksel I. (2020). Polyethylene glycol *versus* split high-dose senna for bowel preparation: A comparative prospective randomized study.. J. Gastroenterol. Hepatol..

[6] Kiambi J., Mwiti C., JN Ngeranwa J., Piero Ngugi M. (2021). Anti-inflammatory potential of dichloromethane leaf extracts of *Eucalyptus globulus* (Labill) and *Senna didymobotrya* (Fresenius) in mice.. Afr. Health Sci..

[7] Arana-Argáez V.E., Domínguez F., Moreno D.A., Isiordia-Espinoza M.A., Lara-Riegos J.C., Ceballos-Góngora E., Zapata-Morales J.R., Franco-De La Torre L., Sánchez-Enríquez S., Alonso-Castro A.J. (2020). Anti-inflammatory and antinociceptive effects of an ethanol extract from *Senna septemtrionalis*.. Inflammopharmacology.

[8] Nadeau S.E., Lawhern R.A. (2022). Management of chronic non-cancer pain: A framework.. Pain Manag. (Lond.).

[9] Oladeji O.S., Adelowo F.E., Oluyori A.P., Bankole D.T. (2020). Ethnobotanical description and biological activities of *Senna alata*.. Evid. Based Compl. Alternat. Med..

[10] Towanou R. (2023). Phytochemical screening, antioxidant activity, and acute toxicity evaluation of *Senna italica* extract used in traditional medicine.. J. Toxicol..

[11] Castro D.T.H., Leite D.F., Da Silva Baldivia D., Dos Santos H.F., Balogun S.O., Da Silva D.B., Carollo C.A., De Picoli Souza K., Dos Santos E.L. (2023). Structural characterization and anticancer activity of a new anthraquinone from *Senna velutina* (Fabaceae).. Pharmaceuticals (Basel).

[12] Roy S., Kundu S., Lyndem L.M. (2016). *Senna* leaf extracts induced Ca^+2^ homeostasis in a zoonotic tapeworm *Hymenolepis diminuta*.. Pharm. Biol..

[13] Li X., Peng X., Qiao B., Peng M., Deng N., Yu R., Tan Z. (2022). Gut-kidney impairment process of adenine ombined with folium sennae-induced diarrhea: association with interactions between *Lactobacillus intestinalis*, *Bacteroides acidifaciens* and acetic acid, inflammation, and kidney function.. Cells.

[14] Candelli M., Franza L., Pignataro G., Ojetti V., Covino M., Piccioni A., Gasbarrini A., Franceschi F. (2021). Interaction between lipopolysaccharide and gut microbiota in inflammatory bowel diseases.. Int. J. Mol. Sci..

[15] Burini R.C., Anderson E., Durstine J.L., Carson J.A. (2020). Inflammation, physical activity, and chronic disease: An evolutionary perspective.. Sports Med. Health Sci..

[16] Guarize L., Da Costa J.C., Dutra L.B., Mendes R.F., Lima I.V.A., Scio E. (2012). Anti-inflammatory, laxative and intestinal motility effects of *Senna macranthera* leaves.. Nat. Prod. Res..

[17] Salguero M., Al-Obaide M., Singh R., Siepmann T., Vasylyeva T. (2019). Dysbiosis of Gram-negative gut microbiota and the associated serum lipopolysaccharide exacerbates inflammation in type-2 diabetic patients with chronic kidney disease.. Exp. Ther. Med..

[18] Li J., Sun Y., Yang N., Zhang H., Hu Y., Wang H., Zhang R., Ge M. (2023). Protective effects of maternal administration of total saponins of Codonopsis pilosula in the mice offspring following diarrhea: role of immune function, antioxidant function, and intestinal inflammatory injury.. Environ. Sci. Pollut. Res. Int..

[19] Zhao T., Wang Z., Liu Z., Xu Y. (2021). Pivotal role of the interaction between herbal medicines and gut microbiota on disease treatment.. Curr. Drug Targets.

[20] Paramita P P., Sajeli B A., Ameer B S., Araya H., Fujimoto Y. (2023). New natural pro-inflammatory cytokines (TNF-α, IL-6 and IL-1β) and iNOS inhibitors identified from Penicillium polonicum through *in vitro* and *in vivo* studies.. Int. Immunopharmacol..

[21] Wang J., Zhang C., Guo C., Li X. (2019). Chitosan ameliorates DSS-induced ulcerative colitis mice by enhancing intestinal barrier function and improving microflora.. Int. J. Mol. Sci..

[22] Furman D., Campisi J., Verdin E., Carrera-Bastos P., Targ S., Franceschi C., Ferrucci L., Gilroy D.W., Fasano A., Miller G.W., Miller A.H., Mantovani A., Weyand C.M., Barzilai N., Goronzy J.J., Rando T.A., Effros R.B., Lucia A., Kleinstreuer N., Slavich G.M. (2019). Chronic inflammation in the etiology of disease across the life span.. Nat. Med..

[23] Mohammad S., Thiemermann C. (2021). Role of metabolic endotoxemia in systemic inflammation and potential interventions.. Front. Immunol..

[24] Gesualdo L., Di Leo V., Coppo R. (2021). The mucosal immune system and IgA nephropathy.. Semin. Immunopathol..

[25] Bai Y., Huang F., Zhang R., Ma Q., Dong L., Su D., Chi J., Zhang M. (2020). Longan pulp polysaccharide protects against cyclophosphamide-induced immunosuppression in mice by promoting intestinal secretory IgA synthesis.. Food Funct..

[26] Li Y., Jin L., Chen T. (2020). The effects of secretory IgA in the mucosal immune system.. Biomed. Res. Int..

[27] Li S., Liang T., Zhang Y., Huang K., Yang S., Lv H., Chen Y., Zhang C., Guan X. (2021). Vitexin alleviates high-fat diet induced brain oxidative stress and inflammation *via* anti-oxidant, anti-inflammatory and gut microbiota modulating properties.. Free Radic. Biol. Med..

[28] Zhang Y., Li Y., Feng Q., Shao M., Yuan F., Liu F. (2020). Polydatin attenuates cadmium-induced oxidative stress *via* stimulating SOD activity and regulating mitochondrial function in *Musca domestica* larvae.. Chemosphere.

[29] Di Meo S., Venditti P. (2020). Evolution of the knowledge of free radicals and other oxidants.. Oxid. Med. Cell Longev..

[30] Ghonimi N.A.M., Elsharkawi K.A., Khyal D.S.M., Abdelghani A.A. (2021). Serum malondialdehyde as a lipid peroxidation marker in multiple sclerosis patients and its relation to disease characteristics.. Mult. Scler. Relat. Disord..

[31] Alizadeh M., Kheirouri S. (2019). Curcumin reduces malondialdehyde and improves antioxidants in humans with diseased conditions: a comprehensive meta-analysis of randomized controlled trials.. Biomedicine (Taipei).

[32] Cecerska-Heryć E., Surowska O., Heryć R., Serwin N., Napiontek-Balińska S., Dołęgowska B. (2021). Are antioxidant enzymes essential markers in the diagnosis and monitoring of cancer patients – A review.. Clin. Biochem..

[33] Zeng Z., He X., Li C., Lin S., Chen H., Liu L., Feng X. (2021). Oral delivery of antioxidant enzymes for effective treatment of inflammatory disease.. Biomaterials.

[34] Krzystek-Korpacka M., Kempiński R., Bromke M.A., Neubauer K. (2020). Oxidative stress markers in inflammatory bowel diseases: systematic review.. Diagnostics (Basel).

[35] Djuricic I., Calder P.C. (2021). Beneficial outcomes of Omega-6 and Omega-3 polyunsaturated fatty acids on human health: an update for 2021.. Nutrients.

[36] Fanelli G., Belardo A., Savino R., Rinalducci S., Zolla L. (2020). Testosterone replacement therapy in insulin-sensitive hypogonadal men restores phosphatidylcholine levels by regulation of arachidonic acid metabolism.. J. Cell. Mol. Med..

[37] Siddiqui M.K., Smith G., St Jean P., Dawed A.Y., Bell S., Soto-Pedre E., Kennedy G., Carr F., Wallentin L., White H., Macphee C.H., Waterworth D., Palmer C.N.A. (2022). Diabetes status modifies the long-term effect of lipoprotein-associated phospholipase A2 on major coronary events.. Diabetologia.

[38] Moreira V., Leiguez E., Janovits P.M., Maia-Marques R., Fernandes C.M., Teixeira C. (2021). Inflammatory effects of bothrops phospholipases A(2): mechanisms involved in biosynthesis of lipid mediators and lipid accumulation.. Toxins (Basel).

[39] Spisni E., Petrocelli G., Imbesi V., Spigarelli R., Azzinnari D., Donati Sarti M., Campieri M., Valerii M.C. (2020). Antioxidant, anti-inflammatory, and microbial-modulating activities of essential oils: implications in colonic pathophysiology.. Int. J. Mol. Sci..

[40] Sztolsztener K., Chabowski A., Harasim-Symbor E., Bielawiec P., Konstantynowicz-Nowicka K. (2020). Arachidonic acid as an early indicator of inflammation during non-alcoholic fatty liver disease development.. Biomolecules.

[41] Amaral E.P., Vinhaes C.L., Oliveira-De-Souza D., Nogueira B., Akrami K.M., Andrade B.B. (2021). The interplay between systemic inflammation, oxidative stress, and tissue remodeling in tuberculosis.. Antioxid. Redox Signal..

[42] He J., Zhang P., Shen L., Niu L., Tan Y., Chen L., Zhao Y., Bai L., Hao X., Li X., Zhang S., Zhu L. (2020). Short-chain fatty acids and their association with signalling pathways in inflammation, Glucose and lipid metabolism.. Int. J. Mol. Sci..

[43] Suzuki R., Mishima M., Nagane M., Mizugaki H., Suzuki T., Komuro M., Shimizu T., Fukuyama T., Takeda S., Ogata M., Miyamoto T., Aihara N., Kamiie J., Kamisuki S., Yokaryo H., Yamashita T., Satoh T. (2023). The novel sustained 3-hydroxybutyrate donor poly-D-3-hydroxybutyric acid prevents inflammatory bowel disease through upregulation of regulatory T-cells.. FASEB J..

[44] Zhang M., Wang Y., Zhao X., Liu C., Wang B., Zhou J. (2021). Mechanistic basis and preliminary practice of butyric acid and butyrate sodium to mitigate gut inflammatory diseases: a comprehensive review.. Nutr. Res..

[45] Zhang L., Qi Y., ALuo Z., Liu S., Zhang Z., Zhou L. (2019). Betaine increases mitochondrial content and improves hepatic lipid metabolism.. Food Funct..

[46] Wang C., Ma C., Gong L., Dai S., Li Y. (2021). Preventive and therapeutic role of betaine in liver disease: A review on molecular mechanisms.. Eur. J. Pharmacol..

[47] Arumugam M.K., Paal M.C., Donohue T.M., Ganesan M., Osna N.A., Kharbanda K.K. (2021). Beneficial effects of betaine: a comprehensive review.. Biology (Basel).

[48] Meng D., Sommella E., Salviati E., Campiglia P., Ganguli K., Djebali K., Zhu W., Walker W.A. (2020). Indole-3-lactic acid, a metabolite of tryptophan, secreted by *Bifidobacterium longum* subspecies infantis is anti-inflammatory in the immature intestine.. Pediatr. Res..

[49] Wei G.Z., Martin K.A., Xing P.Y., Agrawal R., Whiley L., Wood T.K., Hejndorf S., Ng Y.Z., Low J.Z.Y., Rossant J., Nechanitzky R., Holmes E., Nicholson J.K., Tan E.K., Matthews P.M., Pettersson S. (2021). Tryptophan-metabolizing gut microbes regulate adult neurogenesis *via* the aryl hydrocarbon receptor.. Proc. Natl. Acad. Sci. USA.

[50] Pernomian L., Duarte-Silva M., De Barros Cardoso C.R. (2020). The aryl hydrocarbon receptor (AHR) as a potential target for the control of intestinal inflammation: insights from an immune and bacteria sensor receptor.. Clin. Rev. Allergy Immunol..

[51] Puccetti M., Pariano M., Borghi M., Barola C., Moretti S., Galarini R., Mosci P., Ricci M., Costantini C., Giovagnoli S. (2021). Enteric formulated indole-3-carboxaldehyde targets the aryl hydrocarbon receptor for protection in a murine model of metabolic syndrome.. Int. J. Pharm..

[52] Bock K.W. (2020). Aryl hydrocarbon receptor (AHR) functions: Balancing opposing processes including inflammatory reactions.. Biochem. Pharmacol..

[53] Xu X., Sun S., Liang L., Lou C., He Q., Ran M., Zhang L., Zhang J., Yan C., Yuan H., Zhou L., Chen X., Dai X., Wang B., Zhang J., Zhao J. (2021). Role of the aryl hydrocarbon receptor and gut microbiota-derived metabolites indole-3-acetic acid in sulforaphane alleviates hepatic steatosis in mice.. Front. Nutr..

[54] Davidson M., Rashidi N., Hossain M.K., Raza A., Nurgali K., Apostolopoulos V. (2023). Tryptophan and substance abuse: mechanisms and impact.. Int. J. Mol. Sci..

[55] Chen Z., Lin Y., Zhou Q., Xiao S., Li C., Lin R., Li J., Chen Y., Luo C., Mo Z. (2022). Ginsenoside Rg1 mitigates morphine dependence *via* regulation of gut microbiota, tryptophan metabolism, and serotonergic system function.. Biomed. Pharmacother..

[56] Huang T., Zhao L., Lin C.Y., Lu L., Ning Z.W., Hu D.D., Zhong L.L.D., Yang Z.J., Bian Z.X. (2020). Chinese herbal medicine (MaZiRenWan) improves bowel movement in functional constipation through down-regulating oleamide.. Front. Pharmacol..

[57] Kałużna-Czaplińska J., Gątarek P., Chirumbolo S., Chartrand M.S., Bjørklund G. (2019). How important is tryptophan in human health?. Crit. Rev. Food Sci. Nutr..

[58] Gu Z., Pei W., Shen Y., Wang L., Zhu J., Zhang Y., Fan S., Wu Q., Li L., Zhang Z. (2021). *Akkermansia muciniphila* and its outer protein Amuc_1100 regulates tryptophan metabolism in colitis.. Food Funct..

[59] Peredo-Lovillo A., Romero-Luna H.E., Jiménez-Fernández M. (2020). Health promoting microbial metabolites produced by gut microbiota after prebiotics metabolism.. Food Res. Int..

[60] Gostner J.M., Geisler S., Stonig M., Mair L., Sperner-Unterweger B., Fuchs D. (2020). Tryptophan metabolism and related pathways in psychoneuroimmunology: the impact of nutrition and lifestyle.. Neuropsychobiology.

[61] Xue C., Li G., Zheng Q., Gu X., Shi Q., Su Y., Chu Q., Yuan X., Bao Z., Lu J., Li L. (2023). Tryptophan metabolism in health and disease.. Cell Metab..

[62] Correia A.S., Vale N. (2022). Tryptophan metabolism in depression: a narrative review with a focus on serotonin and kynurenine pathways.. Int. J. Mol. Sci..

[63] Höglund E., Øverli Ø., Winberg S. (2019). Tryptophan metabolic pathways and brain serotonergic activity: a comparative review.. Front. Endocrinol. (Lausanne).

[64] Gallegos A., Isseroff R.R. (2022). Simultaneous determination of tryptophan, 5-hydroxytryptophan, tryptamine, serotonin, and 5-HIAA in small volumes of mouse serum using UHPLC-ED.. MethodsX.

[65] Zhang Z.W., Gao C.S., Zhang H., Yang J., Wang Y.P., Pan L.B., Yu H., He C.Y., Luo H.B., Zhao Z.X., Zhou X.B., Wang Y.L., Fu J., Han P., Dong Y.H., Wang G., Li S., Wang Y., Jiang J.D., Zhong W. (2022). *Morinda officinalis* oligosaccharides increase serotonin in the brain and ameliorate depression *via* promoting 5-hydroxytryptophan production in the gut microbiota.. Acta Pharm. Sin. B.

[66] Fukuwatari T. (2020). Possibility of amino acid treatment to prevent the psychiatric disorders *via* modulation of the production of tryptophan metabolite kynurenic acid.. Nutrients.

[67] Dehhaghi M., Kazemi S P H., Guillemin G.J. (2019). Microorganisms, tryptophan metabolism, and kynurenine pathway: A complex interconnected loop influencing human health status.. Int. J. Tryptophan Res..

[68] Chen L.M., Bao C.H., Wu Y., Liang S.H., Wang D., Wu L.Y., Huang Y., Liu H.R., Wu H.G. (2021). Tryptophan-kynurenine metabolism: A link between the gut and brain for depression in inflammatory bowel disease.. J. Neuroinflammation.

[69] Hughes T.D., Güner O.F., Iradukunda E.C., Phillips R.S., Bowen J.P. (2022). The kynurenine pathway and kynurenine 3-monooxygenase inhibitors.. Molecules.

[70] Chen Y., Zhang J., Yang Y., Xiang K., Li H., Sun D., Chen L. (2022). Kynurenine-3-monooxygenase (KMO): From its biological functions to therapeutic effect in diseases progression.. J. Cell. Physiol..

